# S04-3: Reflexive feedback on the use of a local health-enhancing policy audit tool: the CAPLA-santé

**DOI:** 10.1093/eurpub/ckae114.213

**Published:** 2024-09-26

**Authors:** Antoine Noël Racine, Aurélie Van Hoye, Anne Vuillemin

**Affiliations:** Université Côte d’Azur, Lamhess, France; PEMAC, Université de Lorraine, Nancy, France; Université Côte d’Azur, Lamhess, France

## Abstract

**Purpose:**

The implementation of effective intersectoral policies is a key issue in developing an upstream approach to the promotion of health-enhancing physical activity (HEPA). In the last few years, comprehensive frameworks and tools for the analysis of HEPA policies have been developed. In 2018, a local policy audit analysis tool “CAPLA-Santé” was developed by the French Public Health Society with a research team and policy makers from different sectors. Since then, this tool has been used for practical and research purposes. The aim of this project is to collect and analyze feedback on the use of CAPLA-Santé from policy-makers.

**Methods:**

This project involves the capitalization of the experience of CAPLA-Santé users in order to analyze their experiential knowledge in practical application. The users were contacted from the database of the French Public Health Society, which manages the tool. All known users were contacted and invited to take part in an interview. General information about the type of user and the context of use of the tool were collected, and participants were asked to give their reflective feedback on their experience of using the tool.

**Results:**

Elected officials (n = 4) and department heads (n = 4) from two mid-sized French municipalities (n = 2) were interviewed to provide reflective feedback on their experience using CAPLA-Santé. According to the department heads, the tool was useful in promoting discussion and collaboration among different sectors, particularly in achieving better alignment of policies to reach a common goal or shared population target. According to elected officials, CAPLA-Santé was particularly useful for taking stock and formulating a more comprehensive and coherent policy, especially in preparation of a new political mandate. However, the use of the tool required expertise and was seen as time-consuming, which could be a barrier for its use among other municipalities.

**Conclusions:**

A reflexive analysis of the use of the CAPLA-Santé tool, based on the experiences of policy makers and researchers in practice, could help to provide a more comprehensive overview of the needs and perspectives in this field.

**Funding Source:**

No funding

